# Using host-mimicking conditions and a murine cutaneous abscess model to identify synergistic antibiotic combinations effective against *Pseudomonas aeruginosa*

**DOI:** 10.3389/fcimb.2024.1352339

**Published:** 2024-05-14

**Authors:** Nikita Lyons, Weihui Wu, Yongxin Jin, Iain L. Lamont, Daniel Pletzer

**Affiliations:** ^1^ Department of Microbiology and Immunology, School of Biomedical Sciences, University of Otago, Dunedin, New Zealand; ^2^ Department of Biochemistry, School of Biomedical Sciences, University of Otago, Dunedin, New Zealand; ^3^ Department of Microbiology, College of Life Sciences, Nankai University, Tianjin, China

**Keywords:** antibiotic synergy, checkerboard, skin infection model, high-density, host-mimicking conditions, abscess model, drug combinations

## Abstract

Antibiotic drug combination therapy is critical for the successful treatment of infections caused by multidrug resistant pathogens. We investigated the efficacy of β-lactam and β-lactam/β-lactamase inhibitor combinations with other antibiotics, against the hypervirulent, ceftazidime/avibactam resistant *Pseudomonas aeruginosa* Liverpool epidemic strain (LES) B58. Although minimum inhibitory concentrations *in vitro* differed by up to eighty-fold between standard and host-mimicking media, combinatorial effects only marginally changed between conditions for some combinations. Effective combinations *in vitro* were further tested in a chronic, high-density murine infection model. Colistin and azithromycin demonstrated combinatorial effects with ceftazidime and ceftazidime/avibactam both *in vitro* and *in vivo*. Conversely, while tobramycin and tigecycline exhibited strong synergy *in vitro*, this effect was not observed *in vivo*. Our approach of using host-mimicking conditions and a sophisticated animal model to evaluate drug synergy against bacterial pathogens represents a promising approach. This methodology may offer insights into the prediction of combination therapy outcomes and the identification of potential treatment failures.

## Graphical Abstract

Lyons et al. show that antibiotic synergy testing in microbiological growth medium and tissue culture medium result in similar, but not identical, combinatorial effects. Combinatorial effects identified during *in vitro* experiments under host-mimicking conditions were a good, but not perfect, predictor of effects in an *in vivo* murine infection model. The authors’ approach could improve the predictive power of synergy studies at a time when combinatorial therapy is becoming more critical to address antibiotic resistant pathogens.

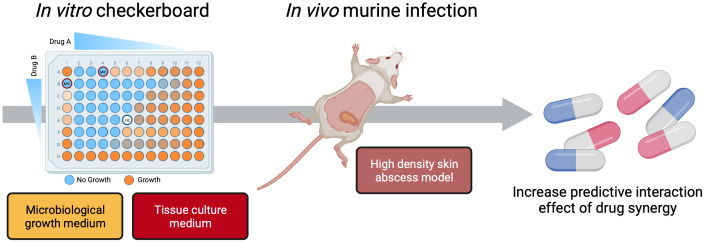

## Highlights

- Antibiotic synergy in host-mimicking tissue culture medium shows similar trends to that in microbiological growth medium although antibiotic susceptibility is reduced
- Antibiotic combinations can restore susceptibility to ceftazidime in ceftazidime-resistant *P. aeruginosa*
- The predictive potential of synergy is enhanced by including host mimicking conditions and should be included for drug synergy evaluation *in vitro*
- Synergistic killing effects under host mimicking conditions suggests the clinical use of ceftazidime/avibactam rather than ceftazidime for improved antimicrobial efficacy
- A combination of *in vitro* assays and an *in vivo* mouse model to improve success of combinatorial therapy and to broaden options to tackle antibiotic resistant pathogens

## Introduction

*Pseudomonas aeruginosa* is an opportunistic gram-negative pathogen which commonly colonises chronic wounds and the lungs of people with cystic fibrosis (CF) (Glen and Lamont, 2021). Hospitalised patients with ventilators, catheters or other long-term indwelling devices are at high risk of contracting nosocomial *P. aeruginosa* infections. Such infections are difficult to treat as *P. aeruginosa* is intrinsically resistant to many antibiotics due to the upregulation of efflux pumps, low outer membrane permeability, target modification, upregulation of chromosomally encoded β-lactamases and acquisition of β-lactamases through horizontal gene transfer (Poole, 2011; Botelho et al., 2019).

First-line treatment for *P. aeruginosa* infections often includes β-lactam antibiotics in combination with a β-lactamase inhibitor (Diaz Santos et al., 2022). β-lactam antibiotics include penicillins, carbapenems, cephalosporins and monobactams that share a common structure (β-lactam ring) and all target penicillin-binding proteins (PBPs) involved in the synthesis of the peptidoglycan layer (Vollmer and Holtje, 2004). *P. aeruginosa* possesses eight PBPs that play important roles in cell division, cross-linking of peptidoglycan peptide chains, and peptidoglycan recycling (Vollmer et al., 2008). β-lactam antibiotics are structural mimics of D-alanine-D-alanyl residues of the peptidoglycan pentapeptide precursor and covalently bind to the active site of PBPs, inactivating them and causing cell wall lysis. The presence of β-lactamases including penicillinase, carbapenemase and cephalosporinase can confer resistance to one or more classes of β-lactams antibiotics (Glen and Lamont, 2021). β-lactamase inhibitors such as avibactam can overcome resistance conferred by certain β-lactamases. Avibactam is a covalent reversible inhibitor for most β-lactamase enzymes (Bush and Bradford, 2016) (class A, C and some D) and unlike another β-lactamase inhibitor, clavulanate (Weber and Sanders, 1990), does not induce expression of β-lactamases (Miossec et al., 2013). Avibactam can be used in combination with ceftazidime (Papp-Wallace, 2019), a cephalosporin β-lactam antibiotic, potentially allowing effective ceftazidime treatment of infections by *P. aeruginosa* that would otherwise be resistant (Nichols et al., 2018).

Treatment of *P. aeruginosa* infections commonly involves an empirically determined combination of a β-lactam/β-lactamase inhibitor drug with an aminoglycoside, fluoroquinolone, or polymyxin depending on the site of infection, patient characteristics and local epidemiology (Bassetti et al., 2018). Although the effectiveness of combinatorial treatment over monotherapy is controversial, with a lack of enough studies on whether it improves clinical outcomes, it is evident that inadequate empirical antibiotic therapy increases mortality (Traugott et al., 2011; Pletzer and Hancock, 2018; Zakhour et al., 2022). Antibiotic combinations can act synergistically, with each antibiotic enhancing the other’s efficacy to produce an inhibitory effect greater than the sum of their individual effects (Fantin and Carbon, 1992; Pletzer et al., 2018). This enhanced effect may be sufficient to restore susceptibility to one or both antibiotics in an otherwise resistant strain. A challenge in predicting antibiotic effectiveness, either alone or in combination, is that *in vitro* susceptibility under standard testing conditions often does not predict treatment effectiveness in a patient (Stratton, 2006; Somayaji et al., 2019; Vazquez-Pertejo, 2022). Lack of correlation can be due to a plethora of differences including nutrient availability, the number of bacteria, drug exposure of bacteria (time and concentration), involvement of the immune system, as well as pharmacodynamic drug effects (from drug absorption, distribution, and metabolism to excretion) occurring in an individual (Stratton, 2006).

Indeed, *in vitro* cultures under more physiologically relevant conditions can substantially alter efficacy across a range of antimicrobials and pathogens (Ersoy et al., 2017; Yung et al., 2021; Vyas et al., 2022). Host mimicking media including eukaryotic cell or tissue culture medium, and addition of blood or serum to standard laboratory media have been developed to replicate conditions of *in vivo* infections more closely (Cantor, 2019). For example, Dulbecco’s Modified Eagle Medium (DMEM) and Roswell Park Memorial Institute (RPMI) medium that are commonly used to culture mammalian cells contain salts, amino acids and vitamins at concentrations mimicking the *in vivo* extracellular milieu. These are also typically supplemented with foetal bovine serum (FBS), providing proteins and growth factors (Shah, 1999). The fundamental differences between laboratory conditions and the infectious environment have recently been explored (Cornforth et al., 2018; Ibberson and Whiteley, 2019). The transcriptome of *P. aeruginosa* cultured in host mimicking conditions is more similar to those collected from wounds than those grown in conventional culture medium (Belanger et al., 2020; Belanger et al., 2022) and antibiotic susceptibility testing under host-mimicking conditions is a more accurate predictor of efficacy or resistance *in vivo* (Ersoy et al., 2017; Sweeney et al., 2020; Belanger and Hancock, 2021; Heithoff et al., 2023).

Given the substantial influence of culture conditions on efficacy of antibiotics, their interactions and synergy may well be altered under physiologically relevant conditions and *in vivo* but this possibility has not been systematically explored. Here, synergy of ceftazidime and ceftazidime/avibactam with other clinically relevant antibiotics were evaluated against *P. aeruginosa* in standard and host-mimicking laboratory media and in a murine high density skin infection model. The combinatorial effects were similar, but not identical, in both media and could, at least partly, predict the effectiveness in the skin infection model. Our study emphasizes the importance of using appropriate methods, including host-mimicking conditions to test drug synergy *in vitro*, and a complex animal model to evaluate drug combination *in vivo*, to tackle antibiotic resistant pathogens.

## Results

### Antibiotics have reduced activity under host-mimicking conditions *in vitro*

Antimicrobial susceptibility testing in conventional microbial culture media such as the EUCAST-recommended Mueller-Hinton medium (EUCAST, 2023), can show poorer predictive accuracy for *in vivo* efficacy than testing in tissue culture medium (TCM) that better represents conditions encountered during infection (Ersoy et al., 2017; Heithoff et al., 2023). Thus, we first determined whether the efficacy of various antibiotics from different classes, against the clinical cystic fibrosis isolate *P. aeruginosa* LESB58, is altered in TCM.

The efficacy of azithromycin increased by 100-fold under host mimicking conditions in tissue culture medium (TCM) compared to standard susceptibility testing medium (MHB), whereas almost all other antibiotics lost efficacy with four to over 80-fold increases in MIC in TCM (**Table 1**). Ceftazidime (CAZ), a strong anti-pseudomonal drug, had a small MIC increase of four-fold in TCM, while the MIC of ceftazidime in combination with the β-lactamase inhibitor avibactam (CZA) was unchanged. Notably, colistin (CST; MIC of 3.12 μg/mL), one of the only antibiotics to which LESB58 is susceptible in MHB based on EUCAST breakpoints (4 μg/mL) (EUCAST, 2023), showed a four-fold increase in MIC bringing it over the clinical susceptibility breakpoint. Aztreonam (ATM) and ciprofloxacin (CIP), another two commonly prescribed anti-pseudomonal drugs, showed a striking eight- and 16-fold increase in MIC in TCM, respectively. Additionally, tobramycin (TOB) and gentamicin (GEN) had a pronounced loss of efficacy showing a 20-fold increase in MIC under host-mimicking conditions. Tigecycline (TGC), a member of glycylcyclines and derivate of tetracycline, is a relative new drug used in critically ill patients for complicated infections where other antibiotics have ceased to work. Worryingly, tigecycline entirely lost its efficacy under host-mimicking conditions against *P. aeruginosa* LESB58. Similar trends were observed in the commonly used laboratory strain *P. aeruginosa* PAO1 (**Supplementary Table S1**).

**Table 1:** Antimicrobial susceptibility of various antibiotics against *P. aeruginosa* LESB58 in two different media.

Drug	ATM^b^	CAZ	CZA^c^	CIP	CST	GEN	TOB	TGC	AZM
MHB (μg/mL)	62.5	31.25	31.25	3.13	3.13	25	3.13	6.25	62.5
TCM (μg/mL)	500	125	31.25	50	12.5	500	62.5	>500	0.625
Fold change TCM vs MHB	8	4	1	16	4	20	20	>80	-100
CLSI breakpoint <=S (μg/mL)^a^	8	8	8	0.5	–	–	1	–	–

a Clinical & Laboratory Standards Institute Guidelines breakpoint based on MIC in MHB; S, sensitive.

b ATM, aztreonam; CAZ, ceftazidime; CZA, ceftazidime/avibactam; CIP, ciprofloxacin; CST, colistin; GEN, gentamicin; TOB, tobramycin; TGC, tigecycline; AZM, azithromycin.

c CZA was used with a 1:4 (AVI : CAZ) ratio, while the clinical breakpoint was determined with a fixed avibactam concentration of 4 mg/L.

Minimum inhibitory concentrations were determined in MHB or TCM after 20-24 h and fold changes calculated between the two media.

-, represents a blank or missing value in the data, indicating that no meaningful data or calculation can be provided for that particular entry or variable.

To delineate whether the higher ceftazidime MIC in TCM was caused by the base medium (DMEM) or by the proteins and growth factors in FBS, we tested the MIC of ceftazidime in MHB supplemented with FBS, and in DMEM in which FBS was replaced by MHB (**Supplementary Table S2**). The MIC of ceftazidime increased eight-fold in MHB when FBS was present and four-fold in TCM containing FBS but only two-fold in TCM where FBS was substituted with MHB (**Supplementary Table S2**). Collectively these findings indicate that FBS is the primary reason for the ceftazidime MIC being higher in TCM than in MHB.

The ceftazidime/avibactam combination had a lower MIC than ceftazidime alone in TCM though not in MHB (**Table 1**; **Supplementary Table S2**). Unexpectedly, avibactam alone had anti-pseudomonal activity in TCM, although this effect was not observed in MHB (**Supplementary Table S2**).

### Antibiotics can act synergistically with ceftazidime to restore susceptibility *in vitro*

Ceftazidime and ceftazidime-avibactam are widely used to treat *P. aeruginosa* infections but many isolates including *P. aeruginosa* LESB58 are resistant to these treatments (Bassetti et al., 2018; Garcia et al., 2022). We investigated whether other clinically relevant antibiotics could act synergistically with ceftazidime or ceftazidime-avibactam, to restore their effectiveness. Synergy is defined as an at least four-fold decrease in MIC of both drugs in combination (fractional inhibitory concentration index [FICI] 0.5), compared to either drug alone. Under standard testing conditions (MHB) ceftazidime synergised with all non-β-lactam antibiotics (**Table 2**) and the combinations reduced ceftazidime MIC to the clinical susceptibility breakpoint of 8 μg/mL or below. Combinations with ceftazidime-avibactam in MHB showed similar trends, except that azithromycin did not act synergistically (**Supplementary Table S3**).

**Table 2:** Synergy experiments with ceftazidime (CAZ) combined with various antibiotics against *P. aeruginosa* LESB58 in two different growth media.

Drug combined with CAZ:		ATM	AVI	CIP	CST	GEN	TOB	TGC	AZM^b^
FICI^a^	MHB	0.75	n.d.^c^	0.5	0.38	0.25	0.38	0.38	0.5
	TCM^d^	n.d.	0.38	0.63	0.38	n.d.	0.38	n.d.	0.625
Fold decrease in CAZ MIC	MHB	2	–	4	4	8	4	4	4
	TCM	–	8	8	8	–	8	–	8
Fold decrease in other MIC	MHB	4	–	4	8	8	8	8	4
	TCM	–	4	2	4	–	4	–	2

a FICI, Fractional inhibitory concentration index (FICI <= 0.5 indicates synergy).

b Synergy with AZM in MHB was determined in *P. aeruginosa* LESB58-lux.

c n.d., not determined.

d Synergy in TCM was determined in *P. aeruginosa* LESB58-lux.

-, represents a blank or missing value in the data, indicating that no meaningful data or calculation can be provided for that particular entry or variable.

We next determined if combinatorial effects were affected by using host-mimicking conditions (culture in TCM instead of MHB) for a subset of antibiotics (**Table 2**; **Supplementary Table S3**). In TCM, colistin and tobramycin synergised with ceftazidime but combined effects with azithromycin or ciprofloxacin were only additive (FICI between 0.5 and 1). Intriguingly, ceftazidime in combination with synergising antibiotics was generally more effective (8-fold decrease in ceftazidime MIC) in TCM compared to MHB (**Table 2**). Ceftazidime-avibactam also acted synergistically, reducing the MIC of both antibiotics by at least four-fold (**Supplementary Table S3**). However, in contrast to the situation with ceftazidime, only colistin synergised with ceftazidime-avibactam under host mimicking conditions, with three other tested combinations showing weaker combinatorial effects in TCM than in MHB (**Supplementary Table S3**). Intriguingly, the combination with ciprofloxacin, although not synergistic in TCM, reduced the ceftazidime-avibactam MIC from 32 μg/mL to 4 μg/mL (8-fold) bringing it below the clinical susceptibility breakpoint (**Supplementary Table S3**). These findings highlighted the potential synergistic therapeutic effects of ceftazidime in combination with colistin or tobramycin, and of ceftazidime-avibactam in combination with colistin. Intriguingly, combinations with ciprofloxacin could revert susceptibility to ceftazidime-avibactam.

### Colistin and azithromycin show synergistic effects with ceftazidime and ceftazidime/avibactam *in vivo*

**Figure 1:**
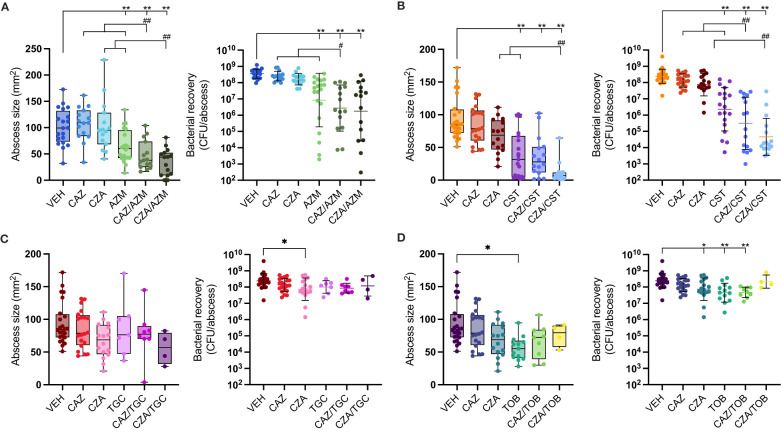
Antibiotic single and combinatorial therapies in a murine subcutaneous abscess model. *P. aeruginosa* LESB58 was injected subcutaneously and treated one hour later with either vehicle, single antibiotics or an antibiotic combination. Abscess sizes are shown as box and whisker plots (left panel) and counted bacteria recovered from the abscess tissue as CFU/abscess with geometric mean and geometric standard deviation (right panel). Combinatorial treatment of ceftazidime (CAZ; 50 mg/mL) or ceftazidime/avibactam (CZA; 50 mg/mL) with **(A)** azithromycin (AZM; 6.25 mg/mL), **(B)** colistin (CST; 1.25 mg/mL), **(C)** tigecycline (TGC; 2.5 mg/mL), or **(D)** tobramycin (TOB; 2 mg/mL). A-D. VEH for AZM experiments was 1.56 mg/mL citric acid in 0.9% saline at pH 7-7.2; VEH for all other experiments was 0.9% saline. VEH, CAZ & CZA mice are shared for CST, TGC & TOB graphs. All experiments were done at least three times with 3-5 mice (6-8 week, female Swiss Webster)/group. Statistical analysis was performed using One-way ANOVA, Kruskal Wallace test with Dunn’s correction (two-sided). Limit of detection for CFU counts was 10^2^. Asterisks indicate significant effect of treatment compared to vehicle control (*p < 0.05; **p < 0.01), and hashes indicate a significant effect of the combination therapy over the sum of the effects of each agent alone (^#^ *p* < 0.05; ^##^ *p* < 0.01).

Although testing antibiotic efficacy in TCM mimics the extracellular milieu of the host environment (Belanger et al., 2022), it does not provide the complex interactions with the host immune system that occur during infection. Thus, we tested ceftazidime and ceftazidime-avibactam in combination with four other antibiotics in a murine subcutaneous abscess model of high-density infection with *P. aeruginosa* LESB58. *P. aeruginosa* LESB58 is resistant to ceftazidime and therefore an excellent isolate for testing whether antibiotic combinations can restore drug susceptibility *in vivo*. Antibiotics were chosen based on their synergistic activity under both laboratory (MHB) and host-mimicking (TCM) media conditions (colistin and tobramycin) or just MHB (azithromycin and tigecycline). All antibiotics were empirically tested *in vivo* (sub-cutaneous) to determine a non-toxic concentration (**Supplementary Table S4**) that reduced skin abscess sizes and/or the bacterial load and would allow us to observe synergy of the drug combinations. Synergy is defined as the combinatorial treatment having a significantly greater effect than the sum of the individual treatments (**Figure 1**) (Fantin and Carbon, 1992; Pletzer et al., 2018). As expected, ceftazidime or ceftazidime-avibactam treatment, even at concentrations as high as 2.5 mg/injection did not by themselves significantly reduce abscess sizes or alter bacterial load compared to vehicle-treated animals (**Figure 1**; **Supplementary Table S5**). Other single drug treatments (azithromycin, colistin and tobramycin) significantly reduced bacterial CFU relative to vehicle-treated animals, by ~42-, 107-, and 5-fold, respectively. They also significantly reduced abscess sizes relative to vehicle-treated mice by 36%, 59%, and 41%. Tigecycline showed no anti-abscess or antimicrobial effect at the highest tolerated dose (**Supplementary Table S5**).

When azithromycin was combined with ceftazidime, abscess sizes were reduced by over 50% and bacterial load reduced by 129-fold compared to the vehicle control (**Figure 1**). Ceftazidime acted synergistically with azithromycin, showing a significant improvement compared to the sum of the individual single treatment. The combination of azithromycin with ceftazidime-avibactam also showed a significant (66%) reduction of abscess sizes, but killing efficacy was only additive although bacterial load was reduced 199-fold compared to the vehicle control (**Figure 1A**).

The highest activity occurred when ceftazidime or ceftazidime-avibactam was combined with colistin, reducing bacterial load by 784- and 5302-fold, respectively, compared to the vehicle control. This activity was synergistic, reducing bacterial load by 60- and 244-fold for ceftazidime and ceftazidime-avibactam respectively compared to the sum of the individual single treatments. This combination also reduced mean abscess sizes by 62% for ceftazidime and 88% for ceftazidime-avibactam relative to the vehicle control (**Figure 1B**; **Supplementary Table S5**).

Conversely, the combinations of tobramycin or tigecycline with ceftazidime or ceftazidime-avibactam showed no further effect beyond those of individual antibiotics on either bacterial load or abscess size (**Figures 1C, D**). This was despite the fact that both antibiotics showed strong synergy (FIC <0.4) with ceftazidime and ceftazidime-avibactam, respectively, in standard laboratory media. The synergistic effect of tobramycin was retained with ceftazidime but not with ceftazidime-avibactam in TCM (**Table 2**; **Supplementary Table S2**). Tigecycline could not be tested for synergy in TCM as it lost its activity (**Table 1**), which was also evident in the mouse infection model (**Figure 1C**).

## Discussion

Antimicrobial susceptibility tests have been widely used to determine the efficacy of a standardized concentration of antimicrobial drugs and drug combinations. However, standard susceptibility testing in regular growth media is increasingly recognized as being a poor predictor of effective treatment *in vivo* (Doern and Brecher, 2011; Ersoy et al., 2017; Heithoff et al., 2023). In fact, drug susceptibility tests often fail to predict clinical efficacy, and resistance in experimental tests does not always predict treatment failure (Doern and Brecher, 2011). Nowadays, combination therapy is frequently used to restore or increase the efficacy of antibiotics against drug resistant strains (El Solh and Alhajhusain, 2009; Tamma et al., 2012). We therefore sought to investigate whether there is also a mismatch between antibiotic combinations tested *in vitro* and their effectiveness *in vivo*. To do this, we determined the synergy of commonly used anti-pseudomonal drugs in a standard growth medium and under more physiologically relevant (host-mimicking) conditions in the laboratory, and in a murine infection model. Although antibiotics have been tested for susceptibility in host mimicking conditions, potential drug interactions under these conditions have not been investigated. To the best of our knowledge, this is the first study to investigate drug combinations under more physiological conditions *in vitro* and a complex, non-lethal murine infection model. Our approach might be a first step into improved prediction of drug combination success or antibiotic combinatorial treatment failure.

We chose ceftazidime, with and without avibactam, as a model drug against *P. aeruginosa*. Synergy occurred with almost all tested antibiotics in MHB, with similar trends in TCM although in several cases the effects were additive rather than synergistic (**Table 2**; **Supplementary Table S3**). Nonetheless, in TCM, all combinations reduced ceftazidime MIC by 8-fold to well below the clinical breakpoint value (**Tables 1**, **2**). Surprisingly, this was not so evident for the ceftazidime/avibactam combinations, although combination with ciprofloxacin and colistin still reduced the ceftazidime/avibactam MIC below the clinical breakpoint (**Table 1**; **Supplementary Table S3**). Interestingly, while ceftazidime showed an increased MIC in TCM compared with MHB, the MIC of the ceftazidime/avibactam combination did not change between the two media. Avibactam alone showed no antimicrobial activity in MHB, as expected, but had significant antimicrobial activity in TCM (**Supplementary Table S2**). Avibactam can bind *P. aeruginosa* PBPs 2 and 3 (Coleman, 2011; Asli et al., 2016; Rajavel et al., 2021). Our data suggest that the lower MIC of the ceftazidime/avibactam combination than ceftazidime alone in TCM may be partly due to the direct effect of avibactam on these PBPs, in addition to its β-lactamase inhibitory activity. This possibility warrants further work into the mechanism of avibactam, but was outside the scope of our study.

Although combinatorial effects between antibiotics were similar in MHB and TCM, MIC values were quite different (**Table 1**). *P. aeruginosa* showed increased susceptibility to one macrolide antibiotic, azithromycin, while being more resistant to β-lactams, aminoglycosides, tetracyclines and polymyxin antibiotics in TCM. These findings are consistent with previous studies (Buyck et al., 2012; Ersoy et al., 2017; Belanger et al., 2020) and emphasize the importance of considering growth conditions during drug evaluation studies. Antibiotic combinations, even if additive rather than synergistic, can restore antibiotic susceptibility in drug resistant strains as shown here and by others (Tait et al., 2021). But regardless of the synergistic or additive effect of antibiotic combinations *in vitro*, they do not account for the host immune system and its important role in individual patients. To further improve and strengthen the predictive value of our *in vitro* synergy studies, we evaluated combinations in a complex, non-lethal, long-term high-density skin infection model. This model (Pletzer et al., 2017) proved well suited to identifying combinatorial and synergistic effects between antibiotics.

Azithromycin had negligible anti-pseudomonal activity under standard laboratory conditions, but was highly effective in host mimicking conditions and *in vivo* (**Table 1**; **Figure 1**), consistent with previous work (Buyck et al., 2012; Belanger et al., 2020; Sorensen et al., 2020). While ceftazidime and azithromycin showed synergy under standard conditions, only additive effects were found in host mimicking conditions. Yet, *in vivo* combinatorial synergistic effects were observed, reducing abscess lesions and bacterial load (**Figure 1**; **Supplementary Table S5**) and emphasising the value of the *in vivo* infection model. Azithromycin has immunomodulatory activity (Cramer et al., 2017) which may contribute to its *in vivo* combinatorial activity with ceftazidime. Combination therapy of azithromycin with ceftazidime is predicted to be effective in our study, as also suggested by others (Fernandez-Cuenca et al., 2003; Wang et al., 2016). Conversely, tigecycline, which also has immunomodulatory activity (Garrido-Mesa et al., 2018), had no effect *in vivo* consistent with its lack of antimicrobial efficacy *in vitro* in TCM, but in contrast to its effects in MHB. Similarly, although synergy of tobramycin with ceftazidime occurred in both standard laboratory medium and TCM (**Table 2**) its antimicrobial activity was reduced by 20-fold in host mimicking conditions (**Table 1**). The loss of lower antimicrobial activity in TCM reflected the *in vivo* situation where the highest tolerated dose had only a small effect on clearing the infection (**Figure 1D**). Lastly, combinations of ceftazidime and colistin have been investigated for over 20 years (Gunderson et al., 2003), and several studies support their combinatorial activity (Mikhail et al., 2019; Mataraci Kara et al., 2020). Our findings support the observation that colistin exhibits strong synergy with both ceftazidime and ceftazidime-avibactam, as demonstrated *in vitro* under standard and host mimicking conditions (**Table 2**; **Supplementary Table S3**), as well as *in vivo* (**Figure 1B**). Nephrotoxicity is a critical side effect of colistin treatment, necessitating dose limitations in patients. Therefore, combining ceftazidime with colistin holds promise for reducing its renal toxicity. Testing drug combinations in physiologically relevant conditions and employing non-lethal animal models could enhance diagnostic accuracy and should be considered in research on effective combinatorial therapies.

This research addresses an important topic that focuses on the discrepancies between various *in vitro* studies and *in vivo* outcomes, with translation into clinical outcomes still in its infancy. Our pathway for drug combinatorial testing, from using MHB to host-mimicking conditions (TCM) to a high-density murine infection model, lays the foundation to optimize drug combinations in the future. We suggest that through combination testing in physiologically relevant media and a relevant infection model, the predictive accuracy of combinatorial drug therapy will be increased.

## Methods

### Strains and growth conditions

*P. aeruginosa* LESB58 (Cheng et al., 1996), *P. aeruginosa* LESB58.lux (Pletzer et al., 2018) or *P. aeruginosa* PAO1 (Hancock and Carey, 1979) was cultured on tryptic soy or double yeast tryptone (dYT) agar (Fort Richards Laboratories) at 37°C overnight. Liquid cultures were grown in dYT broth at 37°C with shaking at 250 rpm.

For susceptibility testing under standard conditions, bacteria were cultured in Muller Hinton Broth MHB (Fort Richards Laboratories). Host mimicking conditions *in vitro* were simulated using tissue culture medium (TCM) consisting of Dulbecco’s modified Eagle medium (DMEM; Gibco #11965) supplemented with FBS (Moregate Biotech) (5% v/v) and glucose (1% w/v). Further susceptibility testing was also conducted in MHB supplemented with FBS (5% v/v) and DMEM supplemented with glucose (1% w/v) and MHB (10% v/v).

### Antibiotics

The following antibiotics were used in this study: azithromycin (AZM; Selleck Chemicals), aztreonam (ATM; Azactam), ceftazidime (CAZ; Biovision), ciprofloxacin (CIP; Sigma Aldrich), colistin (CST; Saphhire Biosciences), gentamicin (GEN; Biochemica), tigecycline (TGC; Adooq Bioscience) and tobramycin (TOB; US Pharmacopeia). The β-lactamase inhibitor avibactam (AVI; MedChem Express) was used alone or in combination with ceftazidime (ceftazidime/avibactam; CZA).

Azithromycin was dissolved in citric acid (Sigma Aldrich) at a 4:1 w/w ratio in 0.9% sterile saline with pH adjusted to 7.0-7.2 using 3 M NaOH. Other antibiotics were dissolved in sterile water for *in vitro* experiments or in 0.9% sterile saline (Sigma Aldrich) for *in vivo* experiments. Avibactam was used at a fixed 1:4 w/w ratio with ceftazidime. All antibiotic stock solutions were filter sterilized using 0.22 μm PES membrane syringe filters.

### Minimum inhibitory and bactericidal concentration

The minimum inhibitory concentration (MIC) of each antibiotic was determined using the broth microdilution method in 96-well plates. All tests were performed in at least triplicate following the Clinical and Laboratory Standards Institute recommendations (CLSI, 2023). Briefly, antibiotics were two-fold serially diluted in MHB or TCM prior to adding 1 × 10^6^ CFU mL^-1^ bacteria. Plates were incubated at 37°C for 22-24 hours. The MIC was recorded as the lowest antibiotic concentration with no visible growth and the median value of at least three biological replicates reported. For experiments using the LESB58.lux strain, no visible growth was defined as an at least 95% reduction compared to the no antibiotic growth control in luminescence detected using a ClarioStar plate reader.

The minimum bactericidal concentration (MBC) was determined by spotting 5 μL from wells of the MIC plate at or above the MIC onto antibiotic free dYT agar. Plates were incubated at 37°C for another 22-24 hours. The MBC was recorded as the lowest antibiotic concentration with no visible growth on agar plates and the median value reported.

### *In vitro* synergy experiments using the checkerboard assay

The *P. aeruginosa* LESB58.lux strain was used for *in vitro* synergy experiments with azithromycin in MHB and all antibiotic combinations in TCM to determine bacterial growth (as visual inspection and absorbance measurements were unreliable). Synergy was determined using the checkerboard method (Berenbaum, 1978; Pletzer and Hancock, 2018) in at least triplicate. Each antibiotic was two-fold serially diluted in sterile water before transferring to a 96-well plate containing 1 × 10^6^ CFU mL^-1^ bacteria in MHB or TCM. Plates were incubated at 37°C for 22-24 hours. The MIC alone or in combination was recorded as the lowest antibiotic concentration with no visible growth. For experiments using the LESB58.lux strain no visible growth was defined as an at least 95% reduction compared to the no antibiotic growth control in luminescence detected using a ClarioStar plate reader. The fractional inhibitory concentration index (FICI) was calculated as [(MIC_A_ in combination)/MIC_A_] + [(MIC_B_ in combination)/MIC_B_]. FICI values of 0.5 or below indicated synergy, while values above 0.5 up to and including 1 were taken to indicate additivity. FICI values above 1 indicate indifference or antagonism (Odds, 2003).

### Murine high density skin abscess model

Mice used in this study were female Swiss Webster aged approximately 6-7 weeks and sourced from the University of Otago Biomedical Research Facility. All animal experiments were approved by the University of Otago Animal Ethics Committee under protocol number AUP19-125. The subcutaneous abscess infection model was performed as previously described (Pletzer et al., 2017) with a higher inoculum. Briefly, the fur on the back of each mouse was removed by shaving and chemical depilation. Antibiotics were tested for skin toxicity by injecting 50 μL of antibiotic suspension subcutaneously into naïve mice, and subsequent inspection of the skin for inflammation on the next day. *P. aeruginosa* LESB58 was grown to mid-log phase in dYT broth, washed twice in sterile PBS (Gibco, pH 7.4), and resuspended in PBS to ~1 x 10^8^ CFU per 50 μL inoculum. The bacterial suspension (50 μL) was injected subcutaneously into the right side of the dorsum, followed one hour later by 50 μL of antibiotic treatment injected directly into the infected area. Antibiotic treatments were administered at the highest non-toxic concentration (CAZ, CZA, CST, TOB) or a dosage that showed a more than 10-fold reduction in recovered bacteria (AZM, TGC) versus vehicle-treated animals. A minimum of 10-fold reduction was required based on power calculations to predict a group of 10-15 mice and on previous experience with this model (Pletzer et al., 2017; Pletzer et al., 2018) to achieve significance. Combination treatments were administered as a single 50 μL injection containing both antibiotics. The concentration of each antibiotic in the combination was the same as when it was used alone. Progression of the infection was monitored daily, and abscess dimensions were measured on day three using a calliper. On day three, abscess tissue and pus were excised, homogenized in sterile PBS using SPEX SamplePrep 1600 MiniG for five minutes and bacterial CFU counts determined by serial dilution. In this model, synergy was defined as being a significant difference between the combination treated group and the amalgamated group of single drug treated animals (Fantin and Carbon, 1992; Pletzer et al., 2018).

### Statistical analysis

Statistical analysis was performed using GraphPad Prism 9.5.1. Bacterial CFU and abscess size data from *in vivo* experiments was analysed by Kruskal-Wallace test with Dunn’s correction for multiple comparisons. The data was considered significant when *p*-values were below 0.05 or 0.01 as indicated.

## Data availability statement

The original contributions presented in the study are included in the article/**Supplementary Material**. Further inquiries can be directed to the corresponding author.

## Ethics statement

The animal study was approved by Animal Ethics Committee, University of Otago AUP19-125. The study was conducted in accordance with the local legislation and institutional requirements.

## Author contributions

NL: Formal analysis, Methodology, Validation, Visualization, Writing – original draft, Investigation. WW: Conceptualization, Funding acquisition, Project administration, Writing – review & editing. YJ: Conceptualization, Funding acquisition, Project administration, Writing – review & editing. IL: Conceptualization, Funding acquisition, Project administration, Writing – review & editing, Methodology, Resources, Supervision, Writing – original draft. DP: Conceptualization, Funding acquisition, Methodology, Project administration, Resources, Supervision, Writing – original draft, Writing – review & editing, Data curation, Formal analysis, Validation, Visualization.

## Supplementary material

Supplementary Table 1 Minimum inhibitory concentrations of antimicrobials against *P. aeruginosa* PAO1 in MHB or TCM.

Supplementary Table 2 Antimicrobial susceptibility of ceftazidime (CAZ) and ceftazidime/avibactam (CZA) against *P. aeruginosa* LESB58 in different growth media. Minimum inhibitory concentrations and minimum bactericidal concentrations in varying concentrations of MHB, DMEM, and FBS. The fold changes were calculated as comparison of the CAZ or CZA MIC in different media compared to standard testing conditions in MHB.

Supplementary Table 3 Synergy experiments with ceftazidime/avibactam (CZA) combined with various antibiotics against *P. aeruginosa* LESB58 in two different growth media.

Supplementary Table 4 Antibiotic toxicity and tolerated dose injected subcutaneously. Toxicity is defined by visible skin irritation, inflammation, blood vessels, swelling. nd, not determined. C/T, ceftolozane/tazobactam.

Supplementary Table 5 Antibiotic activity in the mouse abscess model. Colistin, tigecycline, and tobramycin were compared to the same vehicle as well ceftazidime and ceftazidime/avibactam treatment groups. Percentage reduction and fold changes are compared to the vehicle control.

## References

[B38] Asli A. Brouillette E. Krause K. M. Nichols W. W. Malouin F. (2016). Distinctive binding of avibactam to penicillin-binding proteins of Gram-negative and Gram-positive bacteria. Antimicrob. Agents Chemother. 60, 752–756. doi: 10.1128/AAC.02102-15 26574008 PMC4750707

[B12] Bassetti M. Vena A. Croxatto A. Righi E. Guery B. (2018). How to manage *Pseudomonas aeruginosa* infections. Drugs Context 7, 212527. doi: 10.7573/dic.212527 29872449 PMC5978525

[B29] Belanger C. R. Dostert M. Blimkie T. M. Lee A. H. Dhillon B. K. Wu B. C.. (2022). Surviving the host: microbial metabolic genes required for growth of *Pseudomonas aeruginosa* in physiologically-relevant conditions. Front. Microbiol. 13. doi: 10.3389/fmicb.2022.1055512 PMC9732424 36504765

[B32] Belanger C. R. Hancock R. E. W. (2021). Testing physiologically relevant conditions in minimal inhibitory concentration assays. Nat. Protoc. 16, 3761–3774. doi: 10.1038/s41596-021-00572-8 34215865

[B28] Belanger C. R. Lee A. H. Pletzer D. Dhillon B. K. Falsafi R. Hancock R. E. W. (2020). Identification of novel targets of azithromycin activity against *Pseudomonas aeruginosa* grown in physiologically relevant media. Proc. Natl. Acad. Sci. U.S.A. 117, 33519–33529. doi: 10.1073/pnas.2007626117 33318204 PMC7777150

[B55] Berenbaum M. C. (1978). A method for testing for synergy with any number of agents. J. Infect. Dis. 137, 122–130. doi: 10.1093/infdis/137.2.122 627734

[B2] Botelho J. Grosso F. Peixe L. (2019). Antibiotic resistance in *Pseudomonas aeruginosa* - Mechanisms, epidemiology and evolution. Drug Resist. Update 44, 100640. doi: 10.1016/j.drup.2019.07.002 31492517

[B7] Bush K. Bradford P. A. (2016). β-lactams and β-lactamase inhibitors: an overview. Cold Spring Harb. Perspect. Med. 6 (8), a025247. doi: 10.1101/cshperspect.a025247 27329032 PMC4968164

[B41] Buyck J. M. Plesiat P. Traore H. Vanderbist F. Tulkens P. M. Van Bambeke F. (2012). Increased susceptibility of *Pseudomonas aeruginosa* to macrolides and ketolides in eukaryotic cell culture media and biological fluids due to decreased expression of *oprM* and increased outer-membrane permeability. Clin. Infect. Dis. 55, 534–542. doi: 10.1093/cid/cis473 22573850

[B24] Cantor J. R. (2019). The rise of physiologic media. Trends Cell Biol. 29, 854–861. doi: 10.1016/j.tcb.2019.08.009 31623927 PMC7001851

[B52] Cheng K. Smyth R. L. Govan J. R. Doherty C. Winstanley C. Denning N.. (1996). Spread of beta-lactam-resistant *Pseudomonas aeruginosa* in a cystic fibrosis clinic. Lancet 348, 639–642. doi: 10.1016/S0140-6736(96)05169-0 8782753

[B54] CLSI (2023). Clinical and laboratory standards institute susceptbility testing. CLSI supplement M100, 33rd Edition.

[B40] Coleman K. (2011). Diazabicyclooctanes (DBOs): a potent new class of non-beta-lactam beta-lactamase inhibitors. Curr. Opin. Microbiol. 14, 550–555. doi: 10.1016/j.mib.2011.07.026 21840248

[B26] Cornforth D. M. Dees J. L. Ibberson C. B. Huse H. K. Mathiesen I. H. Kirketerp-Moller K.. (2018). *Pseudomonas aeruginosa* transcriptome during human infection. Proc. Natl. Acad. Sci. U.S.A. 115, E5125–E5134. doi: 10.1073/pnas.1717525115 29760087 PMC5984494

[B45] Cramer C. L. Patterson A. Alchakaki A. Soubani A. O. (2017). Immunomodulatory indications of azithromycin in respiratory disease: a concise review for the clinician. Postgrad Med. 129, 493–499. doi: 10.1080/00325481.2017.1285677 28116959

[B4] Diaz Santos E. Mora Jimenez C. Del Rio-Carbajo L. Vidal-Cortes P. (2022). Treatment of severe multi-drug resistant *Pseudomonas aeruginosa* infections. Med. Intensiva (Engl Ed) 46, 508–520. doi: 10.1016/j.medine.2022.06.014 35840495

[B35] Doern G. V. Brecher S. M. (2011). The clinical predictive value (or lack thereof) of the results of *in vitro* antimicrobial susceptibility tests. J. Clin. Microbiol. 49, S11–S14. doi: 10.1128/JCM.00580-11

[B37] El Solh A. A. Alhajhusain A. (2009). Update on the treatment of *Pseudomonas aeruginosa* pneumonia. J. Antimicrob. Chemother. 64, 229–238. doi: 10.1093/jac/dkp201 19520717

[B21] Ersoy S. C. Heithoff D. M. Barnes L.t. Tripp G. K. House J. K. Marth J. D.. (2017). Correcting a fundamental flaw in the paradigm for antimicrobial susceptibility testing. EBioMedicine 20, 173–181. doi: 10.1016/j.ebiom.2017.05.026 28579300 PMC5478264

[B33] EUCAST (2023) Breakpoint tables for interpretation of MICs and zone diameters, version 13.1. Available at: https://www.eucast.org/clinical_breakpoints.

[B16] Fantin B. Carbon C. (1992). *In vivo* antibiotic synergism: contribution of animal models. Antimicrob. Agents Chemother. 36, 907–912. doi: 10.1128/AAC.36.5.907 1510412 PMC188745

[B47] Fernandez-Cuenca F. Martinez-Martinez L. Pascual A. Perea E. J. (2003). *In vitro* activity of azithromycin in combination with amikacin, ceftazidime, ciprofloxacin or imipenem against clinical isolates of *Acinobacter baumannii* . Chemotherapy 49, 24–26. doi: 10.1159/000069774 12714805

[B34] Garcia P. Brito B. Alcalde-Rico M. Munita J. M. Martinez J. R. W. Olivares-Pacheco J.. (2022). Acquisition of resistance to ceftazidime-avibactam during infection treatment in *Pseudomonas aeruginosa* through D179Y mutation in one of two *bla*(KPC-2) gene copies without losing carbapenem resistance. Front. Cell Infect. Microbiol. 12. doi: 10.3389/fcimb.2022.981792 PMC9478442 36118031

[B48] Garrido-Mesa J. Rodriguez-Nogales A. Algieri F. Vezza T. Hidalgo-Garcia L. Garrido-Barros M.. (2018). Immunomodulatory tetracyclines shape the intestinal inflammatory response inducing mucosal healing and resolution. Br. J. Pharmacol. 175, 4353–4370. doi: 10.1111/bph.14494 30184260 PMC6240124

[B1] Glen K. A. Lamont I. L. (2021). β-lactam resistance in *Pseudomonas aeruginosa*: current status, future prospects. Pathogens 10 (12), 1638. doi: 10.3390/pathogens10121638 PMC8706265 34959593

[B49] Gunderson B. W. Ibrahim K. H. Hovde L. B. Fromm T. L. Reed M. D. Rotschafer J. C. (2003). Synergistic activity of colistin and ceftazidime against multiantibiotic-resistant *Pseudomonas aeruginosa* in an *in vitro* pharmacodynamic model. Antimicrob. Agents Chemother. 47, 905–909. doi: 10.1128/AAC.47.3.905-909.2003 12604520 PMC149291

[B53] Hancock R. E. Carey A. M. (1979). Outer membrane of *Pseudomonas aeruginosa*: heat- 2-mercaptoethanol-modifiable proteins. J. Bacteriol. 140, 902–910. doi: 10.1128/jb.140.3.902-910.1979 118160 PMC216732

[B30] Heithoff D. M. Barnes V. L. Mahan S. P. Fried J. C. Fitzgibbons L. N. House J. K.. (2023). Re-evaluation of FDA-approved antibiotics with increased diagnostic accuracy for assessment of antimicrobial resistance. Cell Rep. Med. 4, 101023. doi: 10.1016/j.xcrm.2023.101023 37116500 PMC10213814

[B27] Ibberson C. B. Whiteley M. (2019). The *Staphylococcus aureus* transcriptome during cystic fibrosis lung infection. mBio 10 (6), e02774-19. doi: 10.1128/mBio.02774-19 31744924 PMC6867902

[B50] Mataraci Kara E. Yilmaz M. Istanbullu Tosun A. Ozbek Celik B. (2020). Evaluation of the synergy of ceftazidime/avibactam in combination with colistin, doripenem, levofloxacin, tigecycline, and tobramycin against OXA-48 producing Enterobacterales. J. Chemother. 32, 171–178. doi: 10.1080/1120009X.2020.1761172 32375606

[B51] Mikhail S. Singh N. B. Kebriaei R. Rice S. A. Stamper K. C. Castanheira M.. (2019). Evaluation of the synergy of ceftazidime-avibactam in combination with meropenem, amikacin, aztreonam, colistin, or fosfomycin against well-characterized multidrug-resistant *Klebsiella pneumoniae* and *Pseudomonas aeruginosa* . Antimicrob. Agents Chemother. 63 (8), e00779-19. doi: 10.1128/AAC.00779-19 31182535 PMC6658738

[B9] Miossec C. Claudon M. Levasseur P. Black M. T. (2013). The beta-lactamase inhibitor avibactam (NXL104) does not induce ampC beta-lactamase in *Enterobacter cloacae* . Infect Drug Resist. 6, 235–240. doi: 10.2147/IDR.S53874 24348054 PMC3857152

[B11] Nichols W. W. Stone G. G. Newell P. Broadhurst H. Wardman A. MacPherson M.. (2018). Ceftazidime-avibactam susceptibility breakpoints against Enterobacteriaceae and *Pseudomonas aeruginosa* . Antimicrob. Agents Chemother. 62 (11), e02590-17. doi: 10.1128/AAC.02590-17 30061279 PMC6201065

[B56] Odds F. C. (2003). Synergy, antagonism, and what the chequerboard puts between them. J. Antimicrob. Chemother. 52, 1–1. doi: 10.1093/jac/dkg301 12805255

[B10] Papp-Wallace K. M. (2019). The latest advances in beta-lactam/beta-lactamase inhibitor combinations for the treatment of Gram-negative bacterial infections. Expert Opin. Pharmacother. 20, 2169–2184. doi: 10.1080/14656566.2019.1660772 31500471 PMC6834881

[B14] Pletzer D. Hancock R. E. W. (2018). Is synergy the key to treating high-density infections? Future Microbiol. 13, 1629–1632. doi: 10.2217/fmb-2018-0216 30426796

[B17] Pletzer D. Mansour S. C. Hancock R. E. W. (2018). Synergy between conventional antibiotics and anti-biofilm peptides in a murine, sub-cutaneous abscess model caused by recalcitrant ESKAPE pathogens. PloS Pathog. 14, e1007084. doi: 10.1371/journal.ppat.1007084 29928049 PMC6013096

[B43] Pletzer D. Mansour S. C. Wuerth K. Rahanjam N. Hancock R. E. W. (2017). New mouse model for chronic infections by Gram-negative bacteria enabling the study of anti-infective efficacy and host-microbe interactions. MBio 8 (1), e00140-17. doi: 10.1128/mBio.00140-17 28246361 PMC5347345

[B3] Poole K. (2011). *Pseudomonas aeruginosa*: resistance to the max. Front. Microbiol. 2. doi: 10.3389/fmicb.2011.00065 PMC3128976 21747788

[B39] Rajavel M. Kumar V. Nguyen H. Wyatt J. Marshall S. H. Papp-Wallace K. M.. (2021). Structural characterization of diazabicyclooctane β-lactam "Enhancers" in complex with penicillin-binding proteins PBP2 and PBP3 of *Pseudomonas aeruginosa* . mBio 12 (1), e03058-20. doi: 10.1128/mBio.03058-20 33593978 PMC8545096

[B25] Shah G. (1999). Why do we still use serum in the production of biopharmaceuticals? Dev. Biol. Stand 99, 17–22. 10404871

[B19] Somayaji R. Parkins M. D. Shah A. Martiniano S. L. Tunney M. M. Kahle J. S.. (2019). Antimicrobial susceptibility testing (AST) and associated clinical outcomes in individuals with cystic fibrosis: A systematic review. J. Cyst Fibros 18, 236–243. doi: 10.1016/j.jcf.2019.01.008 30709744

[B44] Sorensen M. Khakimov B. Nurjadi D. Boutin S. Yi B. Dalpke A. H.. (2020). Comparative evaluation of the effect of different growth media on *in vitro* sensitivity to azithromycin in multi-drug resistant *Pseudomonas aeruginosa* isolated from cystic fibrosis patients. Antimicrob. Resist. Infect. Control 9, 197. doi: 10.1186/s13756-020-00859-7 33298147 PMC7724801

[B20] Stratton C. W. (2006). *In vitro* susceptibility testing versus *in vivo* effectiveness. Med. Clin. North Am. 90, 1077–1088. doi: 10.1016/j.mcna.2006.07.003 17116437

[B31] Sweeney E. Sabnis A. Edwards A. M. Harrison F. (2020). Effect of host-mimicking medium and biofilm growth on the ability of colistin to kill *Pseudomonas aeruginosa* . Microbiol. (Reading) 166, 1171–1180. doi: 10.1099/mic.0.000995 PMC7819359 33253080

[B42] Tait J. R. Bilal H. Kim T. H. Oh A. Peleg A. Y. Boyce J. D.. (2021). Pharmacodynamics of ceftazidime plus tobramycin combination dosage regimens against hypermutable *Pseudomonas aeruginosa* isolates at simulated epithelial lining fluid concentrations in a dynamic *in vitro* infection model. J. Glob Antimicrob. Resist. 26, 55–63. doi: 10.1016/j.jgar.2021.04.021 34023531

[B36] Tamma P. D. Cosgrove S. E. Maragakis L. L. (2012). Combination therapy for treatment of infections with gram-negative bacteria. Clin. Microbiol. Rev. 25, 450–470. doi: 10.1128/CMR.05041-11 22763634 PMC3416487

[B15] Traugott K. A. Echevarria K. Maxwell P. Green K. Lewis J. S. 2nd (2011). Monotherapy or combination therapy? The *Pseudomonas aeruginosa* conundrum. Pharmacotherapy 31, 598–608. doi: 10.1592/phco.31.6.598 21923444

[B18] Vazquez-Pertejo M. T. (2022) Susceptibility testing. Available online at: https://www.msdmanuals.com/en-nz/professional/infectious-diseases/laboratory-diagnosis-of-infectious-disease/susceptibility-testing#:~:text=Susceptibility%20testing%20occurs%20in%20vitro,not%20always%20predict%20treatment%20outcome.

[B5] Vollmer W. Holtje J. V. (2004). The architecture of the murein (peptidoglycan) in gram-negative bacteria: vertical scaffold or horizontal layer(s)? J. Bacteriol. 186, 5978–5987. doi: 10.1128/JB.186.18.5978-5987.2004 15342566 PMC515156

[B6] Vollmer W. Joris B. Charlier P. Foster S. (2008). Bacterial peptidoglycan (murein) hydrolases. FEMS Microbiol. Rev. 32, 259–286. doi: 10.1111/j.1574-6976.2007.00099.x 18266855

[B22] Vyas H. K. N. Xia B. B. Mai-Prochnow A. (2022). Clinically relevant *in vitro* biofilm models: A need to mimic and recapitulate the host environment. Biofilm 4, 100069. doi: 10.1016/j.bioflm.2022.100069 36569981 PMC9782257

[B46] Wang X. Cai Y. Xing H. Wu W. Wang G. Li L.. (2016). Increased therapeutic efficacy of combination of azithromycin and ceftazidime on *Pseudomonas aeruginosa* biofilm in an animal model of ureteral stent infection. BMC Microbiol. 16, 124. doi: 10.1186/s12866-016-0744-1 27341798 PMC4921005

[B8] Weber D. A. Sanders C. C. (1990). Diverse potential of beta-lactamase inhibitors to induce class I enzymes. Antimicrob. Agents Chemother. 34, 156–158. doi: 10.1128/AAC.34.1.156 2327752 PMC171539

[B23] Yung D. B. Y. Sircombe K. J. Pletzer D. (2021). Friends or enemies? The complicated relationship between *Pseudomonas aeruginosa* and *Staphylococcus aureus* . Mol. Microbiol. 116 (1), 1–15. doi: 10.1111/mmi.14699 33576132

[B13] Zakhour J. Sharara S. L. Hindy J. R. Haddad S. F. Kanj S. S. (2022). Antimicrobial treatment of *Pseudomonas aeruginosa* severe sepsis. Antibiotics (Basel) 11 (10), 1432. doi: 10.3390/antibiotics11101432 PMC9598900 36290092

