# Supplementary material for: Using host-mimicking conditions and a murine cutaneous abscess model to identify synergistic antibiotic combinations effective against Pseudomonas aeruginosa
Source: Front Cell Infect Microbiol. 2024 May 14;14:1352339. doi: 10.3389/fcimb.2024.1352339 (PMC11130353; doi:10.3389/fcimb.2024.1352339)
Supplement: Supplementary Table 1 — Minimum inhibitory concentrations of antimicrobials against P. aeruginosa PAO1 in MHB or TCM. [file Table_1.docx]

**Using host-mimicking conditions and a murine cutaneous abscess model to identify synergistic antibiotic combinations effective against *Pseudomonas aeruginosa***

**Nikita Lyons^1,2^, Weihui Wu^3^, Yongxin Jin^3^, Iain Lamont^2^, and Daniel Pletzer^1*^**

1 Department of Microbiology and Immunology, School of Biomedical Sciences, University of Otago, 9054, Dunedin, New Zealand

2 Department of Biochemistry, School of Biomedical Sciences, University of Otago, 9054, Dunedin, New Zealand

3 Department of Microbiology, College of Life Sciences, Nankai University, Tianjin, China.

* Corresponding author: Daniel Pletzer – email address: [daniel.pletzer@otago.ac.nz](mailto:daniel.pletzer@otago.ac.nz), Tel +64 3 479 7478, Mailing address: University of Otago, 720 Cumberland Street, Dunedin 9054, New Zealand.

**Supplementary Data**

**Table S1**. Minimum inhibitory concentrations of antimicrobials against *P. aeruginosa* PAO1 in MHB or TCM.

| **Drug** | **ATM^b^** | **CAZ** | **CZA** | **CIP** | **CST** | **GEN** | **TOB** | **TGC** | **AZM** |
| --- | --- | --- | --- | --- | --- | --- | --- | --- | --- |
| **MHB (μg/mL)** | 12.5 | 3.13 | 3.13 | 0.156 | 2.5 | 1.56 | 0.625 | 12.5 | 250 |
| **TCM (μg/mL)** | 12.5 | 25 | 6.25 | 1.25 | 25 | 25 | 25 | >500 | <0.78 |
| **Fold change  TCM vs MHB** | 1 | 8 | 2 | 8 | 10 | 16 | 40 | >40 | >-300 |
| **CLSI breakpoint (μg/mL)^a^** | 16 | 8 | 8 | 0.5 | 4 | - | 2 | - | - |

^a^ Clinical & Laboratory Standards Institute Guidelines breakpoint based on MIC in MHB

^b^ ATM, aztreonam; CAZ, ceftazidime; CZA, ceftazidime/avibactam; CIP, ciprofloxacin; CST, colistin; GEN, gentamicin; TOB, tobramycin; TGC, tigecycline; AZM, azithromycin

**Table S2. Antimicrobial susceptibility of ceftazidime (CAZ) and ceftazidime/avibactam (CZA) against *P. aeruginosa* LESB58 in different growth media.** Minimum inhibitory concentrations and minimum bactericidal concentrations in varying concentrations of MHB, DMEM, and FBS. The fold changes were calculated as comparison of the CAZ or CZA MIC in different media compared to standard testing conditions in MHB.

| **Medium** | **MHB** | | | **MHB + 5% FBS** | | **TCM (DMEM + 1% glucose + 5% FBS)** | | | **TCM (DMEM + 1% Glucose + 10% MHB)^a^** | |
| --- | --- | --- | --- | --- | --- | --- | --- | --- | --- | --- |
| **Drug** | **CAZ** | **CZA** | **AVI** | **CAZ** | **CZA** | **CAZ** | **CZA** | **AVI** | **CAZ** | **CZA** |
| **MIC (μg/mL)** | 31.25 | 31.25 | >125 | 250 | 125 | 125 | 31.25 | 31.25 | 62.5 | 31.25 |
| **MBC (μg/mL)** | >500 | >500 | >125 | >500 | >500 | >500 | 125 | 62.5 | 500 | 125 |
| **Fold increase MIC of CAZ or CZA** | - | - | - | 8 | 4 | 4 | 1 | - | 2 | 1 |

^a^ *P. aeruginosa* LESB58 does not grow in TCM without supplementation

**Table S3. Synergy experiments with ceftazidime/avibactam (CZA) combined with various antibiotics against *P. aeruginosa* LESB58 in two different growth media.**

| Drug combined with CZA: | | **ATM** | **CIP** | **CST** | **GEN** | **TOB** | **TGC** | **AZM**^b^ |  |
| --- | --- | --- | --- | --- | --- | --- | --- | --- | --- |
| **FICI**^a^ | MHB | 0.75 | 0.5 | 0.38 | 0.38 | 0.38 | 0.25 | 0.75 |  |
|  | TCM^c^ | n.d.^d^ | 0.63 | 0.5 | n.d. | 0.63 | n.d. | 1 |  |
| **Fold decrease in CZA MIC** | MHB | 2 | 4 | 4 | 8 | 4 | 8 | 4 |  |
|  | TCM | - | 8 | 4 | - | 2 | - | 2 |  |
| **Fold decrease in other MIC** | MHB | 4 | 4 | 8 | 8 | 8 | 8 | 2 |  |
|  | TCM | - | 2 | 4 | - | 8 | - | 2 |  |
| ^a^ FICI = Fractional inhibitory concentration index (FICI <= 0.5 indicates synergy) | | | | | | | | | |
| ^b^ Synergy with AZM in MHB was determined in *P. aeruginosa* LESB58-lux | | | | | | | | | |
| ^c^ Synergy in TCM was determined in *P. aeruginosa* LESB58-lux  ^d^ n.d. = not determined | | | | | | | | | |

**Table S4. Antibiotic toxicity and tolerated dose injected subcutaneously.** Toxicity is defined by visible skin irritation, inflammation, blood vessels, swelling. nd, not determined. C/T, ceftolozane/tazobactam.

| **Drug (mg/ml)** | **ATM** | **CAZ** | **CAZ/AVI** | **AVI** | **CIP** | **CST** | **GEN** | **TOB** | **TGC** | **AZM** | **Ceftolozane** | **C/T** | **Tazobactam** | **MEM** |
| --- | --- | --- | --- | --- | --- | --- | --- | --- | --- | --- | --- | --- | --- | --- |
| **Highest tolerated dose tested** | 25 | 50 | 50 (CAZ) 12.5 (AVI) | 12 | 40 | 2 | 20 | 80 | 12 | 25 | 50 | 10 (C) 2.5 (T) | 25 | 10 |
| **Toxic** | - | 100 | 100 (CAZ) 25 (AVI) | nd | 80 | 5 | 40 | nd | 25 | 50 | nd | nd | nd | 20 |
| **Antibiotic solution**^a^ | DS | S | S | S | S | S | S | S | S | CA | S | S | DS | S |

^a^ DS, 50% DMSO - 50% saline; S, saline; CA, citric acid

**Table S5. Antibiotic activity in the mouse abscess model.** Colistin, tigecycline, and tobramycin were compared to the same vehicle as well ceftazidime and ceftazidime/avibactam treatment groups. Percentage reduction and fold changes are compared to the vehicle control.

| **Treatment** | **Area of dermoncecrosis (mm^2^)** | | |  | **CFU per abscess** | |
| --- | --- | --- | --- | --- | --- | --- |
|  | Mean | +/- SE | % reduction |  | Geometric mean | Fold change |
| **Azithromycin group** |  |  |  |  |  |  |
| vehicle | 101.4 | 7.7 |  |  | 3.6 × 10^8^ |  |
| CAZ | 107.3 | 8.3 | - |  | 2.6 × 10^8^ | -1.4 |
| CZA | 102.9 | 12.6 | - |  | 1.6 × 10^8^ | -2.2 |
| AZM | 64.5 | 7.0 | 36.4 |  | 8.5 × 10^6^ | -42.1 |
| AZM/CAZ | 47.2 | 7.1 | 53.4 |  | 2.8 × 10^6^ | -129.1 |
| AZM/CZA | 34.6 | 6.5 | 65.8 |  | 1.8 × 10^6^ | -198.9 |
| Combined single treatment (AZM and CAZ) | 82.9 | 6.4 | 18.3 |  | 3.6 × 10^7^ | -9.8 |
| Combined single treatment (AZM and CZA) | 81.0 | 7.4 | 20.2 |  | 3.0 × 10^7^ | -11.8 |
|  |  |  |  |  |  |  |
| **Colistin group** |  |  |  |  |  |  |
| vehicle | 93.9 | 6.5 |  |  | 2.4 × 10^8^ |  |
| CAZ | 85.3 | 6.4 | 9.2 |  | 1.3 × 10^8^ | -1.8 |
| CZA | 67.1 | 6.5 | 28.6 |  | 7.5 × 10^7^ | -3.3 |
| CST | 38.6 | 8.1 | 58.9 |  | 2.3 × 10^6^ | -107.4 |
| CST/CAZ | 35.4 | 7.7 | 62.3 |  | 3.1 × 10^5^ | -784.4 |
| CST/CZA | 11.1 | 3.9 | 88.2 |  | 4.6 × 10^4^ | -5302.5 |
| Combined single treatment (CST and CAZ) | 62.5 | 6.3 | 33.4 |  | 1.8 × 10^7^ | -13.3 |
| Combined single treatment (CST and CZA) | 51.6 | 5.8 | 45.1 |  | 1.1 × 10^7^ | -21.7 |
|  |  |  |  |  |  |  |
| **Tigecycline group** |  |  |  |  |  |  |
| vehicle | 93.9 | 6.5 |  |  |  |  |
| CAZ | 85.3 | 6.4 | 9.2 |  |  |  |
| CZA | 67.1 | 6.5 | 28.6 |  |  |  |
| TGC | 86.7 | 16.8 | 7.7 |  | 1.0 × 10^8^ | -2.4 |
| TGC/CAZ | 77.4 | 13.6 | 17.6 |  | 8.5 × 10^7^ | -2.8 |
| TGC/CZA | 56.1 | 12.3 | 40.3 |  | 1.2 × 10^8^ | -2.1 |
| Combined single treatment (TGC and CAZ) | 85.6 | 6.3 | 8.8 |  | 1.2 × 10^8^ | -2.0 |
| Combined single treatment (TGC and CZA) | 73.1 | 6.9 | 22.2 |  | 8.2 × 10^7^ | -3.0 |
|  |  |  |  |  |  |  |
| **Tobramycin group** |  |  |  |  |  |  |
| vehicle | 93.9 | 6.5 |  |  |  |  |
| CAZ | 85.3 | 6.4 | 9.2 |  |  |  |
| CZA | 67.1 | 6.5 | 28.6 |  |  |  |
| TOB | 55.6 | 5.0 | 40.9 |  | 4.5 × 10^7^ | -5.4 |
| TOB/CAZ | 65.8 | 8.5 | 29.9 |  | 4.7 × 10^7^ | -5.1 |
| TOB/CZA | 75.9 | 8.6 | 19.2 |  | 2.2 × 10^8^ | -1.1 |
| Combined single treatment (TOB and CAZ) | 73.6 | 5.0 | 21.7 |  | 8.7 × 10^7^ | -2.8 |
| Combined single treatment (TOB and CZA) | 61.9 | 4.3 | 34.1 |  | 6.0 × 10^7^ | -4.1 |
